# Supplementary material for: Topological protection of multiparticle dissipative transport
Source: Nat Commun. 2016 Jun 1;7:11745. doi: 10.1038/ncomms11745 (PMC4895435; doi:10.1038/ncomms11745)
Supplement: Supplementary Information — Supplementary Figures 1-4 and Supplementary Note 1 [file ncomms11745-s1.pdf]

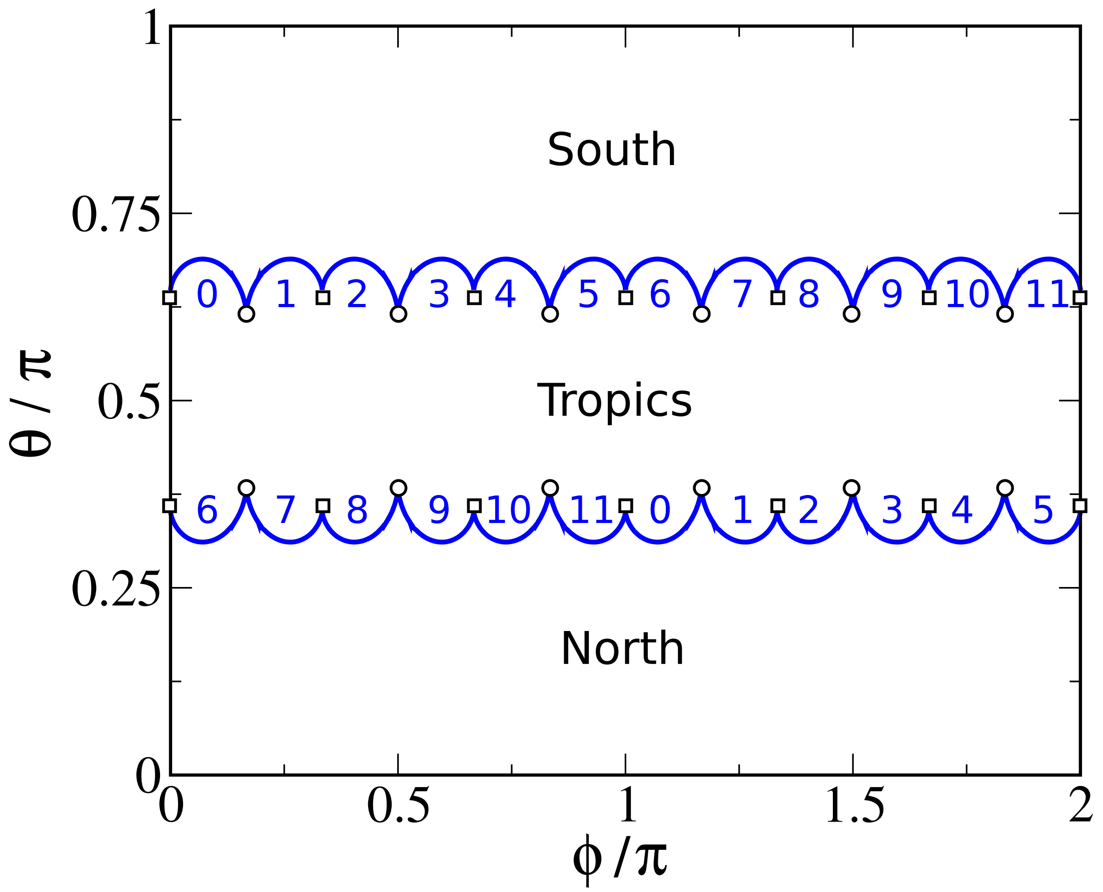

Supplementary Figure 1. **Borders of control space**. The borders of control space are the projections of the fences. Each border contains 12 segments that we label from 0 to 11 as indicated. The projection of $\mathcal{F}^{0+}$ ($\mathcal{F}^{0-}$) is the northern (southern) border and separates the tropics from the north (south) of $\mathcal{C}$. Empty circles are projection of triple zero bifurcation points. Empty squares in the northern (southern) border indicate the projection of triple plus (minus) points.

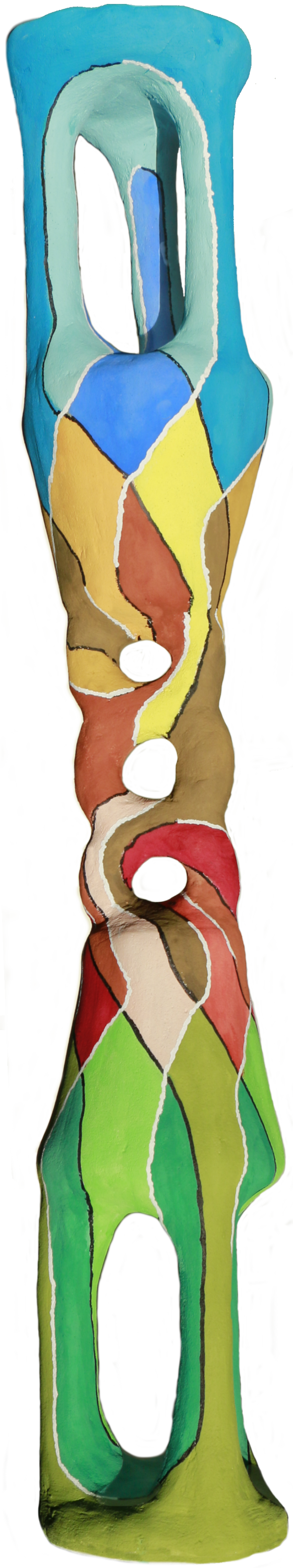

Supplementary Figure 2. **Stationary surface $\mathcal{M}$.** A plaster model of the stationary surface $\mathcal{M}$. Each color is a bijective area. The green (blue) areas represent $\mathcal{M}_+$ ($\mathcal{M}_-$). The remaining areas (earth color tones) form $\mathcal{M}_0$. The black and white lines separating two different areas represent segments of fences or pseudo fences. The color of the line indicates whether the segment is odd (white) or even (black). The stationary manifold $\mathcal{M}$ is actually a two-dimensional surface in the four-dimensional curved space $\mathcal{C} \otimes \mathcal{A}$ that cannot be visualized. We can however embed $\mathcal{M}$ in a Cartesian three-dimensional space, as the figure shows. Both, the real $\mathcal{M}$ in $\mathcal{C} \otimes \mathcal{A}$ and the model shown in the figure are topologically equivalent. That is, both can be continuously deformed into each other.

	0	1	2	3	4	5	6	7	8	9	10	11
t_+	n_{+2}	n_{+2}	n_{+1}	n_{+1}	n_{+2}	n_{+2}	n_{+1}	n_{+1}	n_{+2}	n_{+2}	n_{+1}	n_{+1}
n_{+1}	n_{01}	n_{03}	t_+	t_+	n_{02}	n_{01}	t_+	t_+	n_{03}	n_{02}	t_+	t_+
n_{+2}	t_+	t_+	n_{03}	n_{02}	t_+	t_+	n_{01}	n_{03}	t_+	t_+	n_{02}	n_{01}
n_{01}	n_{+1}	t_{01}	t_{01}	t_{01}	t_{01}	n_{+1}	n_{+2}	t_{02}	t_{02}	t_{02}	t_{02}	n_{+2}
n_{02}	t_{02}	t_{02}	t_{02}	n_{+2}	n_{+1}	t_{01}	t_{01}	t_{01}	t_{01}	n_{+1}	n_{+2}	t_{02}
n_{03}	t_{01}	n_{+1}	n_{+2}	t_{02}	t_{02}	t_{02}	t_{02}	n_{+2}	n_{+1}	t_{01}	t_{01}	t_{01}
t_{01}	n_{03}	n_{01}	n_{01}	n_{01}	n_{01}	n_{02}	n_{02}	n_{02}	n_{02}	n_{03}	n_{03}	n_{03}
t_{02}	n_{02}	n_{02}	n_{02}	n_{03}	n_{03}	n_{03}	n_{03}	n_{01}	n_{01}	n_{01}	n_{01}	n_{02}
n_-	t_-	t_-	t_-	t_-	t_-	t_-	t_-	t_-	t_-	t_-	t_-	t_-
t_-	n_-	n_-	n_-	n_-	n_-	n_-	n_-	n_-	n_-	n_-	n_-	n_-

Supplementary Figure 3. **Neighbouring areas in $\mathcal{M}$.** The top-most row indicates a segment of a fence or a pseudo fence in $\mathcal{M}$. The left-most column is a list of the bijective areas involved in the motion of diamagnets. The inner cells indicate the neighbouring bijective areas. For example, the neighbour of t_+ in segment 1 is n_{+2}. This is a segment of a pseudo fence since both areas t_+ and n_{+2} are on $\mathcal{M}_+$. The neighbour of n_{+1} in segment 9 is n_{02}. In this case the segment belongs to the fence $\mathcal{F}^{0+}$ since it separates areas on $\mathcal{M}_0$ and $\mathcal{M}_+$. A similar table can be constructed for the bijective areas involved in the motion of paramagnets.

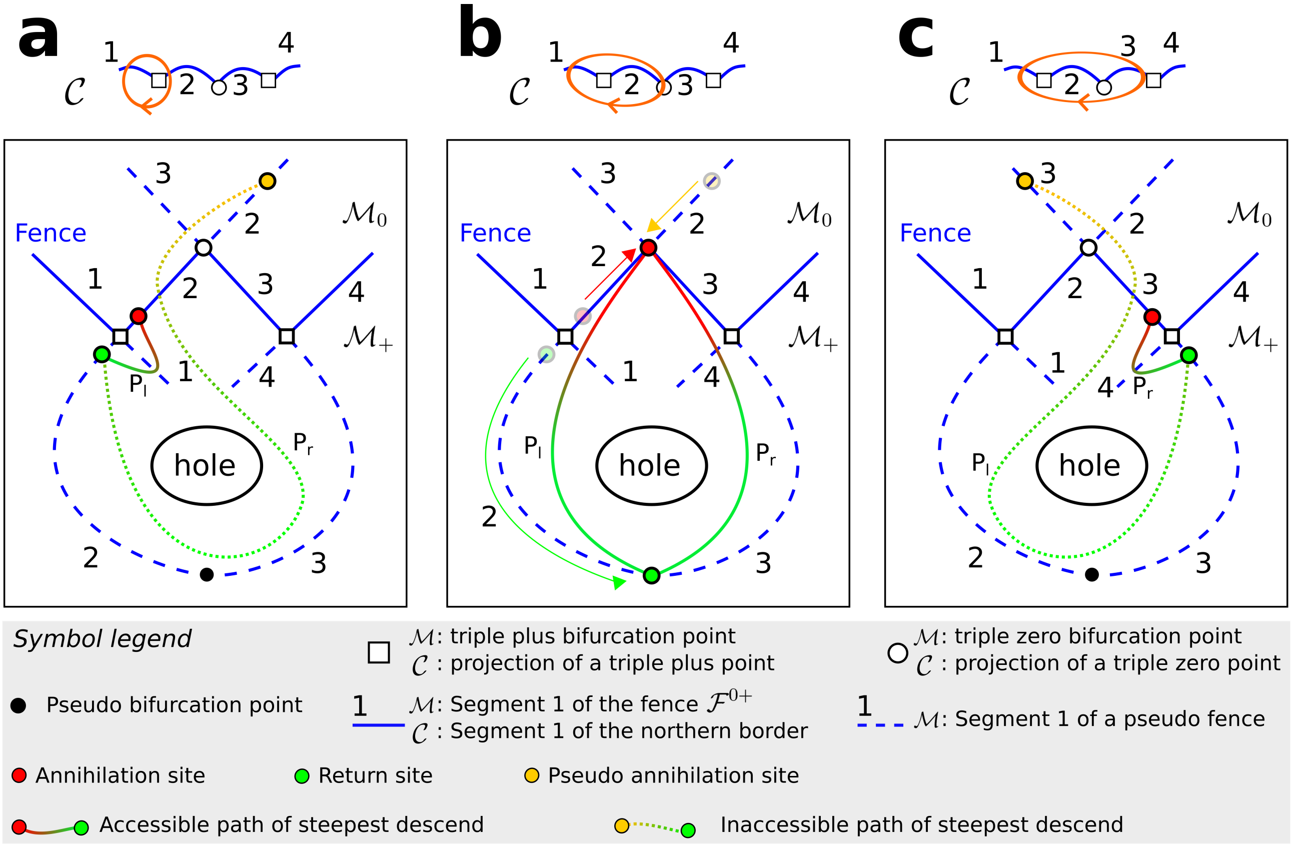

Supplementary Figure 4. **Topological transition of the transport direction**. Ratchet modulation loop (a) $\mathcal{L}_\mathcal{C} = (1_\mathrm{N}, 2_\mathrm{N})$, (b) passing through the projection of a $\mathcal{B}^0$ bifurcation point, and (c) $\mathcal{L}_\mathcal{C} = (1_\mathrm{N}, 3_\mathrm{N})$ (top panels) and their corresponding path of steepest descend connecting points in $\mathcal{M}$ (bottom panels). (a) The accessible path of steepest descend (P_l) connects the annihilation and return sites. The annihilation site is a preimage of the point where $\mathcal{L}_\mathcal{C}$ crosses from the north to the tropics in $\mathcal{C}$. Another preimage of the same point is the pseudo annihilation site, which is also connected to the return site through the path of steepest descend P_r. However, P_r is inaccessible because it develops from $\mathcal{M}_0$. Both P_l and P_r are in $\mathcal{C} \otimes \mathcal{A}$, not in $\mathcal{M}$. They lie on opposite sides of the hole of $\mathcal{C} \otimes \mathcal{A}$, and hence induce transport in different directions. (b) The modulation loop passes through the projection of $\mathcal{B}^0$. The annihilation site has moved (red arrow) along the segment 2 of the fence toward the $\mathcal{B}^0$ bifurcation point. The return site has moved (green arrow) along the segment 2 of the pseudo fence in $\mathcal{M}^+$ toward the pseudo bifurcation point. The pseudo annihilation site has moved (yellow arrow) along the segment 2 of the pseudo fence in $\mathcal{M}^0$ toward the $\mathcal{B}^0$ bifurcation point. Hence, the annihilation and the pseudo-annihilation site merge at the $\mathcal{B}^0$ bifurcation point. Both paths of steepest descend P_l and P_r are at this point accessible and they are topologically distinct. Following one path of steepest descend and returning in opposite direction via the other defines a non-zero homotopic loop that winds around a hole of $\mathcal{C} \otimes \mathcal{A}$. For this $\mathcal{L}_\mathcal{C}$ two ratchets with different directions coexist. (c) $\mathcal{L}_\mathcal{C}$ encircles the projection of $\mathcal{B}^0$, and hence the former annihilation site in (a) has moved along the segment 3 of a pseudo fence in $\mathcal{M}_0$, changing to a pseudo annihilation site. Its corresponding path of steepest descend P_l is now inaccessible. The other path P_r is now accessible, changing the transport direction.

SUPPLEMENTARY NOTE 1

Total external field. The pattern is a hexagonal lattice of bubbles with positive magnetization M immersed in an extended domain of negative magnetization $-M$. The domain walls between regions of opposite magnetization are very sharp. To obtain the total magnetic field $\mathbf{H}$ we solve the Laplace equation $\mathbf{\Delta H} = 0$ subject to the boundary conditions:

$$\begin{aligned} \mathbf{H}(\mathbf{x}_\mathcal{A}, z \to \infty) &= \mathbf{H}_{\text{ext}} \\ H_z(\mathbf{x}_\mathcal{A}, z = 0) &= m(\mathbf{x}_\mathcal{A}), \end{aligned} \tag{1}$$

where the local magnetization $m(\mathbf{x}_\mathcal{A})$ is given by

$$m(\mathbf{x}_\mathcal{A}) = \begin{cases} +M & \text{if } \mathbf{x}_\mathcal{A} \in \text{bubble} \\ -M & \text{if } \mathbf{x}_\mathcal{A} \notin \text{bubble.} \end{cases} \tag{2}$$

The solution, given as a Fourier series, is:

$$\mathbf{H}(\mathbf{x}_\mathcal{A}, z) = \begin{pmatrix} \mathbf{H}^{||}_{\text{ext}} \\ \tilde{H}^z_{\text{ext}} \end{pmatrix} + 2(\tilde{M} + \tilde{H}^z_{\text{ext}}) \sum_{n=0}^{\infty} \sum_{m=0}^{n-1}{}' \frac{J_1(q_{nm}R)}{(q_{nm}R)^2} e^{-(q_{nm}z)} \sum_{j=1}^{6} \begin{pmatrix} \mathbf{R}^{\mathbf{j}}_{\pi/\mathbf{3}} \cdot \mathbf{q}_{nm} R \sin(\mathbf{R}^{\mathbf{j}}_{\pi/\mathbf{3}} \cdot \mathbf{q}_{nm} \cdot \mathbf{x}_\mathcal{A}) \\ q_{nm} R \cos(\mathbf{R}^{\mathbf{j}}_{\pi/\mathbf{3}} \cdot \mathbf{q}_{nm} \cdot \mathbf{x}_\mathcal{A}). \end{pmatrix} \tag{3}$$

The presence of the ferrofluid renormalizes the magnetization and the z component of the external field, $\tilde{M} = M/(1+\chi)$ and $\tilde{H}^z_{\text{ext}} = H^z_{\text{ext}}/(1+\chi)$. In the above expression J_1 is the order one Bessel function of the first kind, $\mathbf{q}_{nm} = n\mathbf{q}^{(1)} + m\mathbf{q}^{(2)}$ is a reciprocal lattice vector with reciprocal unit vectors

$$\mathbf{q}^{(1)} = \frac{2\pi}{a \sin(\pi/3)} \begin{pmatrix} \cos(\pi/6) \\ -\sin(\pi/6) \end{pmatrix} \qquad \mathbf{q}^{(2)} = \frac{2\pi}{a \sin(\pi/3)} \begin{pmatrix} 0 \\ 1 \end{pmatrix}. \tag{4}$$

q_{nm} denotes the magnitude of $\mathbf{q}_{nm}$. The radius of the magnetic bubbles can be found by matching the magnetic flux at $z = 0$ and $z \to \infty$, the result is $R = a\sqrt{(H^z_{ext}/M + 1)\sin(\pi/3)/(2\pi)}$, with a the period of the hexagonal lattice. The prime at the double sum in Supplementary Eq. (3) denotes the exclusion of the zero reciprocal vector ($n = m = 0$) of the first Brillouin zone from the sum. $\mathbf{R}_{\pi/\mathbf{3}}$ is a rotation matrix that rotates all vectors by $\pi/3$ in the plane.

The Fourier modes decay exponentially as one moves away from the garnet film surface. At the elevation of the colloids only the leading order reciprocal lattice vectors of the second Brillouin zone ($n = 1, m = 0$) are needed for an accurate description of the field. The magnetic field at high elevations is therefore given by

$$\mathbf{H}(\mathbf{x}_\mathcal{A}, z >> 0) \approx \begin{pmatrix} \mathbf{H}^{||}_{\text{ext}} \\ \tilde{H}^z_{\text{ext}} \end{pmatrix} + 2(\tilde{M} + \tilde{H}^z_{\text{ext}}) \frac{J_1(q_2 R)}{(q_2 R)^2} e^{-(q_2 z)} \sum_{i=1}^{6} \begin{pmatrix} \mathbf{q}_{2_i} \sin(\mathbf{q}_{2_i} \cdot \mathbf{x}_\mathcal{A}) \\ q_2 \cos(\mathbf{q}_{2_i} \cdot \mathbf{x}_\mathcal{A}) \end{pmatrix}, \tag{5}$$

where the sum runs only over the six reciprocal lattice vectors of the second Brillouin zone

$$\mathbf{q}_{2_i} = \frac{2\pi}{a} \begin{pmatrix} -\sin(2\pi i/6) \\ \cos(2\pi i/6) \end{pmatrix}, \qquad i = 1, .., 6, \tag{6}$$

and $q_2 = 2\pi/a$. The colloids follow the magnetic potential $V_{\text{m}} = -\chi_{\text{eff}}\mu_0 H^2$. We use the unique scaled-potential $V = H^2$ to describe the motion of both diamagnets and paramagnets. At high elevations, the leading (not constant) term of V is given by

$$V \propto \sum_{i=1}^{6} \begin{pmatrix} \mathbf{H}^{||}_{\text{ext}} \\ \tilde{H}^z_{\text{ext}} \end{pmatrix} \cdot \begin{pmatrix} \mathbf{q}_{2_i} \sin(\mathbf{q}_{2_i} \cdot \mathbf{x}_\mathcal{A}) \\ q_2 \cos(\mathbf{q}_{2_i} \cdot \mathbf{x}_\mathcal{A}) \end{pmatrix}. \tag{7}$$
